# Supplementary material for: Cognitive regulation alters social and dietary choice by changing attribute representations in domain-general and domain-specific brain circuits
Source: eLife. 2018 May 29;7:e31185. doi: 10.7554/eLife.31185 (PMC5973829; doi:10.7554/eLife.31185)
Supplement: Supplementary file 1. — (A) Drift diffusion model (DDM) parameters and RTs. Table reports Mean and Standard Deviations (±SD). b = initial height of the barrier; d = rate of decay for collapsing barriers; NDT = non decision time.; RTs = reaction times. Results for the Altruism Task are reported both for full sample (N = 49), and for the subset of participants that also completing the Food Task (N = 36). (B) Support vector regressions (SVRs) of decision values (DV) in both choice tasks. Results for goal-independent encoding of DVs (Main effect for DV, averaged information content across three conditions in each task) are reported at a statistical threshold of p < 0.05, FWE corrected at voxel-level; † indicates results for goal-dependent variations in DV (repeated measures ANOVAs across three conditions in each task) reported at p < 0.001, FWE cluster-corrected at p < 0.05, k = 10 voxels; only peak activations of clusters are reported; L = left hemisphere, R = right hemisphere, k = cluster size in voxels, MNI = Montreal Neurological Institute. (C) Correlation of regulatory success in goal-dependent attribute weighting across choice domains. Correlation coefficients for change scores in attribute weights (w) in food choices and altruistic choices estimated in two separate DDMs. Successful reduction in the weight on selfish considerations (Δw $Self) in altruistic choices was correlated with successfully amplifying the weight on health considerations (Δw Healthiness) suppressing the weight of taste considerations (Δw Tastiness) in food choices. Changes in the ability to increase social considerations (Δw Other) were not correlated to goal-consistent changes in food attributes. Δw Tastiness [NC - TC] is not displayed, as estimated attribute weights did not significantly differ between conditions. Note also that differences scores in Δw Healthiness [NC - TC] (last column) were minimal, limiting the interpretability of the respective correlation analyses. (D) Decoding of individual differences in r [file elife-31185-supp1.docx]

**Supplementary File 1:**

**Supplemental Tables for**

**Cognitive regulation alters social and dietary choice by changing attribute representations in domain-general and domain-specific brain circuits**

Anita Tusche *^1^ and Cendri Hutcherson ^2,3^

^1^ Division of the Humanities and Social Sciences, California Institute of Technology, Pasadena, CA 91125, U.S.A.

^2^ Department of Psychology, University of Toronto Scarborough, Toronto, ON M1C 1A4, Canada

^3^ Department of Marketing, Rotman School of Management, University of Toronto, Toronto, ON M5S 3E6, Canada

Correspondence to: [atusche@caltech.edu](mailto:atusche@caltech.edu)

**Supplementary file 1A. Drift diffusion model (DDM) parameters and RTs.**

Table reports Mean and Standard Deviations (± SD). b = initial height of the barrier; d = rate of decay for collapsing barriers; NDT = non-decision time.; RTs = reaction times. Results for the Altruism Task are reported both for full sample (N = 49), and for the subset of participants that also completing the Food Task (N = 36).

|  |  | Food Task |  |
| --- | --- | --- | --- |
|  | Respond Naturally [NC] | Focus on Health [HC] | Focus on Taste [TC] |
| b (N = 36) | .259 (± .080) | .246 (± .083) | .249 (± .082) |
| d (N = 36) | .001 (± .001) | .001 (± .000) | .001 (± .001) |
| NDT (N = 36) | .438 (± .132) | .493 (± .157) | .474 (± .113) |
| RTs (N = 36) | 1.43 (± .460) | 1.58 (± .493) | 1.46 (± .464) |
|  |  | Altruism Task |  |
|  | Respond Naturally [NC] | Focus on Partner [PC] | Focus on Ethics [EC] |
| b (N = 49) | .258 (± .081) | .291 (± .055) | .284 (±.065) |
| (N = 36) | .251 (± .085) | .300 (± .047) | .281 (± .061) |
| d (N = 49) | .001 (± .001) | .001 (± .001) | .001 (± .001) |
| (N = 36) | .001 (± .001) | .001 (± .001) | .001 (± .001) |
| NDT (N = 49) | .643 (± .235) | .643 (± .238) | .601 (± .202) |
| (N = 36) | .650 (± .242) | .613 (± .218) | .599 (± .211) |
| RTs (N = 49) | 1.81 (± .489) | 1.90 (± .497) | 1.82 (± .497) |
| (N = 36) | 1.80 (± .492) | 1.87 (± .491) | 1.82 (± .496) |

**Supplementary file 1B. Support vector regressions (SVRs) of decision values (DV) in both choice tasks.**

Results for goal-independent encoding of DVs (Main effect for DV, averaged information content across three conditions in each task) are reported at a statistical threshold of p < 0.05, FWE corrected at voxel-level; ^†^ indicates results for goal-dependent variations in DV (repeated measures ANOVAs across three conditions in each task) reported at p < 0.001, FWE cluster-corrected at p < 0.05, k = 10 voxels; only peak activations of clusters are reported; L = left hemisphere, R = right hemisphere, k = cluster size in voxels, MNI = Montreal Neurological Institute.

| Brain region | Side | T | k | MNI | | |
| --- | --- | --- | --- | --- | --- | --- |
|  |  |  |  | x | y | z |
| *Main Effect for DV in Food Task* |  |  |  |  |  |  |
| Medial prefrontal cortex (MPFC) (extends to right DLPFC, right mid frontal gyrus) | L/R | 9.11 | 1736 | -12 | 53 | 37 |
| Precuneus/ visual cortex | L/R | 8.22 | 2119 | 9 | -64 | 52 |
| Inferior parietal cortex | R | 6.71 | 135 | 42 | -64 | 34 |
| Motor cortex | L | 9.90 | 520 | -42 | -19 | 58 |
| *Main Effect for DV in Altruism Task* |  |  |  |  |  |  |
| Motor cortex (extends to bilateral MPFC, left DLPFC, bilateral visual cortex) | L | 11.65 | 7048 | -39 | -25 | 58 |
| Inferior frontal gyrus | L | 6.38 | 290 | -45 | 26 | -2 |
| Inferior frontal gyrus | L | 5.75 | 60 | -45 | 8 | 22 |
| Inferior/mid frontal gyrus | R | 4.49 | 20 | 33 | 59 | 10 |
| DLPFC | R | 6.33 | 84 | 45 | 5 | 25 |
| DLPFC | R | 6.21 | 132 | 36 | 32 | 25 |
| DLPFC | R | 5.21 | 11 | 48 | 5 | 40 |
| Superior temporal gyrus | L | 5.79 | 15 | -63 | -25 | 10 |
| Supramarginal gyrus | R | 5.84 | 48 | 54 | -31 | 40 |
| Supplemental motor area | R | 6.81 | 344 | 6 | -10 | 64 |
| Supplemental motor area | R | 5.80 | 13 | 54 | -4 | 55 |
| *Goal-dependent encoding for DV in Food Task* ^†^ |  |  |  |  |  |  |
| [TC > HC]: Motor cortex | L | 4.47 | 201 | -33 | -37 | 64 |
| *Goal-dependent encoding of DV in Altruism Task ^†^* |  |  |  |  |  |  |
| [EC > PC]: Cerebellum | L | 4.75 | 251 | -27 | -70 | -35 |

**Supplementary file 1C. Correlation of regulatory success in goal-dependent attribute weighting across choice domains.**

Correlation coefficients for change scores in attribute weights (w) in food choices and altruistic choices estimated in two separate DDMs. Successful reduction in the weight on selfish considerations (Δw $Self) in altruistic choices was correlated with successfully amplifying the weight on health considerations (Δw Healthiness} suppressing the weight of taste considerations (Δw Tastiness) in food choices. Changes in the ability to increase social considerations (Δw Other) were not correlated to goal-consistent changes in food attributes. Δw Tastiness [NC - TC] is not displayed, as estimated attribute weights did not significantly differ between conditions. Note also that differences scores in Δw Healthiness [NC - TC] (last column) were minimal, limiting the interpretability of the respective correlation analyses.

| Altruistic Attributes | Food Attributes | | | | |
| --- | --- | --- | --- | --- | --- |
|  | Δw Tastiness  [NC - HC] | Δw Tastiness  [TC - HC] | Δw Healthiness  [HC - NC] | Δw Healthiness  [HC - TC] | Δw Healthiness  [NC - TC] |
| Δw $Self [NC - EC] | 0.25 | 0.27 | *0.33 ^†^* | 0.29 | 0.01 |
| Δw $Self [NC - PC] | ***0.43*** * | ***0.45*** * | ***0.50*** * | ***0.46*** * | 0.08 |
| Δw $Self [EC - PC] | *0.34 ^†^* | *0.35 ^†^* | *0.36 ^†^* | *0.34 ^†^* | 0.10 |
| Δw $Other [EC - NC] | 0.00 | -0.02 | 0.02 | 0.04 | 0.07 |
| Δw $Other [PC - NC] | 0.21 | 0.20 | 0.23 | 0.23 | 0.08 |
| Δw $Other [EC - PC] | -0.27 | -0.28 | -0.27 | -0.24 | -0.01 |
| Δw Fairness [EC - NC] | 0.32 | *0.36* ^†^ | 0.20 | 0.28 | *0.34 ^†^* |
| Δw Fairness [PC - NC] | -0.32 | *-0.38* ^†^ | -0.11 | -0.11 | -0.06 |
| Δw Fairness [EC - PC] | ***0.45 **** | ***0.51*** * | 0.22 | 0.30 | 0.31 |

* p < 0.05 FDR corrected ^[[1]](#footnote-1),^ ^[[2]](#footnote-2)^; ^†^ p ≤ 0.05 uncorrected

**Supplementary file 1D. Decoding of individual differences in regulatory success in DLPFC (altruism task).**

Decoding of individual differences in regulation success based on response patterns in right DLPFC (Figure 5A) obtained in the altruism task. Response patterns reliably predicted the extent of increased generous choice behavior. Consistent with key results reported in the main text, neural activation patterns also predicted individual’s increased healthy choices in a separate food task. Regarding altered attributes weights, predictive information in DLPFC was selective for subjects’ inhibition of $Self weights, but did not extend to altered weights on $Other or Fairness, confirming results reported in the main text. Higher-than-chance predictions are reported when decoding accuracy values exceeded the 95th percentile of empirical null-distribution (cutoff), obtained with 1000 replications of the analysis on permuted data sets.

|  | Decoding accuracy [r] | P Values in Permutation Test |
| --- | --- | --- |
| Δ Generous Choices [NC - (EC, PC)] | 0.55 | 0.010 |
| Δ Healthy Choices [HC - (NC, TC)] | 0.38 | 0.042 |
| Δw $Self [NC - (EC, PC)] | 0.49 | 0.014 |
| Δw $Other [NC - (EC, PC)] | -0.31 | 0.88 |
| Δw Fairness [NC - (EC, PC)] | -0.06 | 0.57 |

**Supplementary file 1E. Univariate encoding of attributes in food task and altruism task.**

Regions reported as significant if they passed a cluster-corrected threshold p < 0.05, with a voxel-defining threshold of p < 0.001, uncorrected, unless otherwise noted. * Illustrates results significant at p < 0.001, uncorrected, reported for completeness: subgenual area did not overlap with the area of vmPFC that displayed overlapping representations of all attributes; only peak activations of clusters are reported; L = left hemisphere, R = right hemisphere, MNI=Montreal Neurological Institute, k = cluster size in voxels.

| Brain region | Side | T | k | MNI | | |
| --- | --- | --- | --- | --- | --- | --- |
|  |  |  |  | x | y | z |
| *Main Effect of Healthiness* - No regions significant |  |  |  |  |  |  |
| *Main Effect of Tastiness* |  |  |  |  |  |  |
| VMPFC | L/R | 8.45 | 450 | -6 | 44 | -5 |
| *Main Effect of $Self* |  |  |  |  |  |  |
| Occipital cortex | L | 5.74 | 211 | -36 | -82 | 7 |
| Insula, Putamen | L | 4.72 | 118 | -36 | -1 | 4 |
| Occipital cortex | R | 4.71 | 130 | 36 | -73 | 1 |
| MPFC/Anterior Cingulate | L/R | 4.45 | 216 | -6 | 38 | 10 |
| Inferior parietal lobule | R | 3.91 | 103 | 45 | -58 | 46 |
| *Main Effect of $Other* - No regions significant. |  |  |  |  |  |  |
| *Main Effect of Fairness* |  |  |  |  |  |  |
| Parietal Cortex | R | 5.18 | 117 | 57 | -37 | 55 |
| *Regulatory Modulation of Healthiness* - No regions significant |  |  |  |  |  |  |
| *Regulatory Modulation of Tastiness* |  |  |  |  |  |  |
| [(TC, NC) > HC] Subgenual Cortex* | L | 3.82 | 28 | -3 | 35 | -5 |
| *Regulatory Modulation of $Self* - No regions significant |  |  |  |  |  |  |
| *Regulatory Modulation of $Other* - No regions significant |  |  |  |  |  |  |
| *Regulatory Modulation of Fairness* - No regions significant |  |  |  |  |  |  |

1. implemented using the fdr_bh function in Matlab [↑](#footnote-ref-1)
2. Benjamini Y, Hochberg Y (1995) Controlling the False Discovery Rate - a Practical and Powerful Approach to Multiple Testing. Journal of the Royal Statistical Society Series B-Methodological 57:289-300.

   Benjamini Y, Yekutieli D (2001) The control of the false discovery rate in multiple testing under dependency. The Annals of Statistics 29:1165-1188. [↑](#footnote-ref-2)
