# Supplementary material for: Cognitive regulation alters social and dietary choice by changing attribute representations in domain-general and domain-specific brain circuits
Source: eLife. 2018 May 29;7:e31185. doi: 10.7554/eLife.31185 (PMC5973829; doi:10.7554/eLife.31185)
Supplement: Transparent reporting form [file elife-31185-transrepform.docx]

***eLife’s* transparent reporting form**

We encourage authors to provide detailed information *within their submission* to facilitate the interpretation and replication of experiments. Authors can upload supporting documentation to indicate the use of appropriate reporting guidelines for health-related research (see [EQUATOR Network](http://www.equator-network.org/%20)), life science research (see the [BioSharing Information Resource](https://biosharing.org/)), or the [ARRIVE guidelines](http://www.plosbiology.org/article/info:doi/10.1371/journal.pbio.1000412) for reporting work involving animal research. Where applicable, authors should refer to any relevant reporting standards documents in this form.

If you have any questions, please consult our Journal Policies and/or contact us: [editorial@elifesciences.org](mailto:editorial@elifesciences.org).

**Sample-size estimation**

- You should state whether an appropriate sample size was computed when the study was being designed
- You should state the statistical method of sample size computation and any required assumptions
- If no explicit power analysis was used, you should describe how you decided what sample (replicate) size (number) to use

Please outline where this information can be found within the submission (e.g., sections or figure legends), or explain why this information doesn’t apply to your submission:

Sample sizes of both established fMRI tasks were based on previous implementation of this task that successfully identified both behavioral and neural effects. References to these studies are included in the respective paragraphs of the method section of the paper (‘Participants’, p. 32).

**Replicates**

- You should report how often each experiment was performed
- You should include a definition of biological versus technical replication
- The data obtained should be provided and sufficient information should be provided to indicate the number of independent biological and/or technical replicates
- If you encountered any outliers, you should describe how these were handled
- Criteria for exclusion/inclusion of data should be clearly stated
- High-throughput sequence data should be uploaded before submission, with a private link for reviewers provided (these are available from both GEO and ArrayExpress)

Please outline where this information can be found within the submission (e.g., sections or figure legends), or explain why this information doesn’t apply to your submission:

Subjects performed each fMRI task once.

A detailed description of criteria for inclusion of subjects and exclusion of behavioral data (invariant choice behavior of one subject in the food task) and neural data (head movement exceeding 3mm/3degree) is provided in the method section of the paper (‘participants’).

Outlier detection: Visual inspection pointed towards one regulatory success score of one subject in the altruism task as potential outlier (difference score of weight put on your own monetary payoff [$Self] in natural and regulation choice conditions) (Figure 4C, D). The data point did not exceed our exclusion criterion of [Mean +/- 3*SD]. However, to confirm the robustness of our findings, we repeated the respective analyses without this data point. The results of these supplemental analyses are provided in the figure legend of Figure 4C and D.

**Statistical reporting**

- Statistical analysis methods should be described and justified
- Raw data should be presented in figures whenever informative to do so (typically when N per group is less than 10)
- For each experiment, you should identify the statistical tests used, exact values of N, definitions of center, methods of multiple test correction, and dispersion and precision measures (e.g., mean, median, SD, SEM, confidence intervals; and, for the major substantive results, a measure of effect size (e.g., Pearson's r, Cohen's d)
- Report exact p-values wherever possible alongside the summary statistics and 95% confidence intervals. These should be reported for all key questions and not only when the p-value is less than 0.05.

Please outline where this information can be found within the submission (e.g., sections or figure legends), or explain why this information doesn’t apply to your submission:

The relevant information is provided in the result section of the manuscript, as well as in supplemental tables. Behavioral raw data are illustrated in Figure 1C,D and Figure 4A-C.

(For large datasets, or papers with a very large number of statistical tests, you may upload a single table file with tests, Ns, etc., with reference to sections in the manuscript.)

**Group allocation**

- Indicate how samples were allocated into experimental groups (in the case of clinical studies, please specify allocation to treatment method); if randomization was used, please also state if restricted randomization was applied
- Indicate if masking was used during group allocation, data collection and/or data analysis

Please outline where this information can be found within the submission (e.g., sections or figure legends), or explain why this information doesn’t apply to your submission:

Subjects performed two separate fMRI tasks as part of a large-scale cross-sectional research project (Conte Center Study at the California Institute of Technology). Task order was fixed, with the food task completed on average 16 months (SD: ± 8.66; range: 1-24) after the altruism task, with the specific goal to probe for common and distinct computations in non-social and social goal-dependent choices. All subjects performed all experimental conditions within a task.

**Additional data files (“source data”)**

- We encourage you to upload relevant additional data files, such as numerical data that are represented as a graph in a figure, or as a summary table
- Where provided, these should be in the most useful format, and they can be uploaded as “Source data” files linked to a main figure or table
- Include model definition files including the full list of parameters used
- Include code used for data analysis (e.g., R, MatLab)
- Avoid stating that data files are “available upon request”

Please indicate the figures or tables for which source data files have been provided:

No source data is provided at his point. Group-level contrast files will be uploaded onto NeuroVault after publication of the manuscript. Behavioral and neural data are part of a large-scale cross-sectional research project (Conte Center Study at the California Institute of Technology) and are part of two other research projects with questions unrelated to the current manuscript (status: under preparation). After publication, behavioral and neural data will be made available on OSF.

The full list of parameters for computational models is provided in the respective methods sections.
